# The associations among athlete gratitude, athlete engagement, athlete burnout: A cross-lagged study in China

**DOI:** 10.3389/fpsyg.2022.996144

**Published:** 2022-09-29

**Authors:** Zhengmao Guo, Jian Yang, Ming Wu, Yatao Xu, Shuang Chen, Shouming Li

**Affiliations:** ^1^School of Physical Education, Shanghai Normal University, Shanghai, China; ^2^Postdoctoral Mobile Station of Physical Education, Wuhan Sports University, Wuhan, China; ^3^College of Physical Education and Health, East China Normal University, Shanghai, China; ^4^School of Physical Education (Main Campus), Zhengzhou University, Zhengzhou, China; ^5^Department of Physical Education, Nanjing University of Finance & Economics, Nanjing, China; ^6^National Physique Monitoring Center, Anhui Institute of Sports Science, Hefei, China

**Keywords:** athlete, gratitude, athlete engagement, athlete burnout, cross-lagged

## Abstract

The purpose of this study is to explore the causal relationship among athlete gratitude, athlete engagement, athlete burnout by cross-lag analysis of longitudinal associations. Two questionnaire surveys were conducted on 352 Chinese athletes with an interval of 1 year using gratitude questionnaire, athlete engagement questionnaire and athlete burnout questionnaire. The analysis yielded four main findings. (1) The overall level of athlete gratitude and athlete engagement was high in China. Chinese athletes at master level had higher levels of gratitude and athlete engagement than athletes at I and II grades. (2) Athlete gratitude is a significant negative predictor of athlete burnout, and also a significant positive predictor of athlete engagement. (3) Athlete engagement and athlete burnout are mutually causal and can be mutually predicted. (4) Athlete gratitude indirectly affects athlete burnout through athlete engagement, and also indirectly affects athlete engagement through athlete burnout. The results of the current study demonstrated the important value of gratitude in the growth process of athletes, and clarified the mechanism of gratitude affecting athlete engagement and athlete burnout. These findings have important implications for athlete development by raising athlete gratitude, motivating athlete engagement levels and relieve athlete burnout.

## Introduction

Gratitude refers to the psychological tendency of individuals to understand or respond the kindness or help of others with grateful cognitions, emotions, and behaviors that lead to positive experiences for themselves (McCullough et al., 2002). Since ancient times, gratitude has been a traditional virtue of the Chinese people and a spiritual support and moral foundation for the inheritance of human civilization and the progress of social development (Dong et al., 2016). With the rise of positive psychology, gratitude has gradually become an important topic in psychological theory and practice. In the field of sports, gratitude is also the focus of core values and quality education cultivation of athletes, and there are too many people and events throughout athletes’ life that are closely related to gratitude, such as coaches’ careful help and guidance to athletes, logisticians’ selfless dedication, athletes’ mutual help and care, and athletes’ participation in social welfare activities. Along with the acceleration of the professionalization and marketization of sports, the cultivation of a sound personality of athletes is an issue worthy of our great attention, and gratitude is an important element of cultivating a sound personality. Thanksgiving words and deeds of athletes not only have a powerful social effect and produce certain social repercussions, but also have a profound impact on their own development. On the one hand, gratitude not only facilitates the development of positive attributions, but also increases athletes’ subjective well-being, eliminates the adverse effects triggered by negative emotions, and builds and maintains athletes’ psychological coping resources (Dong et al., 2016). Research studies by scholars Chen et al. (2008), Ye et al. (2016a) and Yukhymenko-Lescroart et al. (2022) others have found the predictive effect of gratitude on athlete burnout. On the other hand, gratitude, as a positive emotion, can motivate athletes to change their perception of the world and they are more willing to spend time focusing on sports training and competition, and this positive emotion can lead to positive changes in athletes’ behavior, besides, scholars Jiang (2008), Wang et al. (2014), and others found the predictive effect of athlete gratitude on athlete engagement. In addition, domestic and foreign scholars have also explored the relationship between athlete engagement and athlete burnout. Scholars Guo et al. (2021) and others found that athlete engagement has a predictive effect on athlete burnout, while scholars Samienia et al. (2022) and others found that athlete burnout also predicts athlete engagement, meanwhile, psychological capital, psychological empowerment and psychological need have also been found in the fields of psychology and organizational management to be predictive of work engagement (Amor et al., 2021; Niswaty et al., 2021; Liebenberg et al., 2022).

Previous studies have mostly used cross-sectional data to explore the relationship between athlete gratitude, athlete engagement, and athlete burnout, but the studies were lack of tracking data, and the causal relationship between athlete engagement and athlete burnout has not been clarified. Based on this, this study used Chinese professional athletes as subjects to conduct a longitudinal study on athlete gratitude, athlete engagement and athlete burnout using tracking data. Theoretically, this study can deeply explore the relationship among athlete gratitude, athlete engagement, athlete burnout, and further clarify its internal mechanism, which has certain reference value for improving the level of athlete engagement and alleviating athlete burnout. In practice, this study can highlight the importance of gratitude education for athletes, provide scientific support for the in-depth development of gratitude education for athletes, and effectively guide the cultivation of core values of athletes.

## Theory and hypotheses

### Athletes gratitude and athlete burnout

The cognitive-emotional stress model suggests that athlete burnout is a stressful effect of a chronic imbalance between environmental demands and individual resources, and is the final breakdown stage of an athlete’s chronic exposure to undesirable stress (Smith, 1986). Athlete burnout not only causes decreased motivation, increased injuries, and decreased training and life satisfaction (Groenewal et al., 2021; Russell, 2021; Gençay and Gençay, 2022), but it is also an important trigger for athlete’s early retirement (Sarmento et al., 2021). Early researchers examined the stressors that lead to athlete burnout mainly from the perspective of stress (Dale and Weinberg, 1990; Raedeke et al., 2002). In recent years, a positive psychology perspective has emerged as a hot topic of research to explore the “immunity” of athletes to effectively copy with athlete burnout, i.e., which elements of psychological capital can mitigate athlete burnout (Shang and Yang, 2021; Zhang et al., 2021). Based on broaden-and-build theory of positive emotions, Fredrickson proposed that gratitude, as a positive emotion, not only has a broaden effect on cognition but also builds psychological resources. Studies have confirmed that gratitude significantly boosts individuals’ subjective well-being, academic achievement (Kong et al., 2021; Valdez et al., 2022), and reduces stress perception, depression, loneliness, and improves psychological well-being (Moltafet and Sharifi, 2021). In addition, gratitude also improves the quality of sleep and promotes the recovery of health functions in individuals by improving pre-sleep cognition (Wood et al., 2009; Shah, 2021). In the field of sports, scholars Ye et al. (2016a) also confirmed the close association between athlete gratitude and athlete burnout through cross-sectional data of 483 Chinese professional athletes. Based on the above analysis, it can be hypothesized that athlete gratitude can inhibit athlete burnout by increasing athletes’ psychological resources, relieving athletes’ stress, and promoting athletes’ physical and mental balance, whereby it is proposed that:

*Hypothesis 1:* Athlete Gratitude has a significant negative predictive effect on Athlete Burnout.

### Athlete gratitude and athlete engagement

Scholars such as Lonsdale et al. (2007b) pioneered the concept of athlete engagement in the field of competitive sports and defined it as an enduring, positive cognitive and emotional experience in sports, with confidence, dedication, vigor, and enthusiasm as the main characteristics. The exploration of influencing factors and the formation mechanism of athlete engagement provides a favorable opportunity to explore the potential and strengths of athletes. A large number of studies on study engagement and work engagement have been conducted in the current academic community, and the results indicate that social-background factors such as parents, teachers, peers, and leaders, as well as self-relevant variables such as self-efficacy and personal resilience are closely associated with individual study engagement and work engagement (Ye, 2014). In general, individuals who feel more favors from others and show more tendency to be grateful will interpret daily experiences more positively, are more likely to recall positive events, experience the good things in life, better appreciate the kindness given by others, and feel loved and taken care of. Such positive cognitive tendencies can improve individuals’ interaction patterns with the external environment, stimulate their sense of responsibility and dedication, and thus show more pro-social behavior and experience more social support. In the field of education, scholars Wen et al. (2010) found gratitude to be a significant predictor of junior high school study engagement based on a study of 669 Chinese junior high school students. In the field of sports, the positive effect of gratitude also exists, as Chen (2013) found that gratitude positively predicted team satisfaction and life satisfaction in athletes, and also helped to enhance the social support level of coaches and teammates. Intrinsic and extrinsic goal theory suggests that individuals with high levels of gratitude will pursue more intrinsic goals, which can effectively contribute to the satisfaction of basic psychological needs (Yu et al., 2010), furthermore, the level of satisfaction significantly and positively predicts athlete engagement (Hodge et al., 2009). Scholars Wang et al. (2014) also found a strong association between athlete gratitude and athlete engagement based on cross-sectional data from 472 active athletes in China. Based on the above analysis, it can be hypothesized that athlete gratitude can contribute to positive changes in athletes’ behavior by promoting the formation of positive cognitive dispositions, which in turn enhance athletes’ athlete engagement levels, whereby it is proposed that:

*Hypothesis 2:* *Athlete Gratitude has a significant positive predictive effect on Athlete Engagement.*

### Athlete engagement and athlete burnout

Both athlete engagement and athlete burnout are important factors influencing athletic performance of athletes, but the causal relationship between them is unclear. Research in the field of organizational management suggests that work burnout and work engagement are two extreme manifestations of work status, and they are the two poles of a three-dimensional continuum in which engagement is characterized by energy, involvement, and efficacy, which happen to be the direct opposites of the three fatigue dimensions of exhaustion, cynicism, and lack of professional efficacy. Individuals with high engagement feel energetic and are able to fully engage in their work; in contrast, individuals with high fatigue have a sense of low efficacy and depletion and are in a state of detachment from their work and others (Wang and Qin, 2009; Bakker et al., 2014; Dai et al., 2021). In research in the field of sport, there are two main perspectives to explore the relationship between the two. For one, athlete engagement affects athlete burnout. Hodge et al. (2009) and Ye et al. (2016b), and others found that when athletes’ basic psychological needs are met and their cognition, emotion and behavior are in a high state of engagement, they can stimulate athletes’ athletic potential to a greater extent, have more fluid experience, and be more satisfied with their athletic performance. Zhang (2012) also found that athlete engagement is an important indicator of the positive psychological aspects of athletes, and that high levels of athlete engagement are conducive to stimulating positive qualities and alleviating and suppressing negative psychological states of athletes. As early explanatory model of athlete burnout, the sport commitment model, also suggests that individual engagement in sport situations can be effective in alleviating athlete burnout (Scanlan et al., 1993; Schmidt and Stein, 2010). For another, athlete burnout affects athlete engagement. Athlete burnout is of interest to sports scientists because of its negative effects on athletic training and competition. It has been confirmed that athlete burnout can be an important predictor of the satisfaction of training and competition and athletic motivation, and the more psychologically fatigued an athlete is, the lower his or her training satisfaction and athletic motivation are (Zhang, 2010), while athletic motivation is an important predictor of athlete engagement (Li et al., 2020). Although previous studies have explored the intrinsic link between athlete engagement and athlete burnout, there are few studies on the causal tracing of athlete engagement and athlete burnout. Based on the above analysis, it is believed that athlete engagement and athlete burnout may influence each other, and high athlete engagement can stimulate positive psychological qualities of sports and alleviate negative psychological states; conversely, persistent athlete burnout can also reduce athletes’ motivation to play sports, increase athletes’ avoidance tendency and reduce athletes’ athlete engagement. Accordingly, it is proposed that:

*Hypothesis 3:* Athlete Engagement has a significant negative predictive effect on Athlete Burnout.

*Hypothesis 4:* Athlete Burnout has a significant predictive negative effect on Athlete Engagement.

In addition, Ye et al. (2016a) found that there is no significant gender difference in the level of athlete, but there is a significant difference of sports grade in China. The higher of sports grade, the higher the level of gratitude they hold. Song and Wu (2012) took college students as survey objects and found that the gender difference of college students’ sport engagement was significant, and the total score of male students’ sports engagement was significantly higher than that of female students. At present, there are few studies on the gender difference and sports grade difference of professional athletes’ sport engagement. Xia (2019) found that there was no significant gender difference in the psychological fatigue of young athletes, Zhao (2020) found that there is no significant difference between the national grade-1 athletes and the grade-2 athletes in athlete burnout. This study will also systematically examine the gender differences and sports grade differences in athlete gratitude, athlete engagement, athlete burnout.

## Materials and methods

### Participants and procedure

Each sports team shall be taken as a unit for group testing, and the informed consent of the local sports bureau, coaches and athletes shall be obtained before testing. We stated that the investigation was anonymous, and that its results would only be used for scientific research and would not pose any risk to them in their daily life. We also informed them that participation in the research was entirely voluntary. The duration of the test was about 30 min. All the questionnaires were collected by the researchers on the spot. According to local legislation and institutional requirements, there is no need for the study to undergo ethical review or obtain signed informed consent forms from participants. However, a process to ensure that all participants have informed consent to participate is still included in the survey. The method of protecting the privacy of the subjects has been presented in the Supplementary materials.

Athletes from several professional sports teams in Heilongjiang, Shandong, Shanghai, Anhui, Hubei and Sichuan provinces and cities were selected as subjects for questionnaire survey and signed informed consent forms. Affected by the training and competition cycle of athletes and the COVID-19 epidemic, the follow-up interval used in this study was 1 year. The first test (T1) was conducted from September 2020, and the second follow-up test (T2) was conducted from September 2021. A total of 476 valid subjects were obtained in the first survey and 352 valid subjects in the second time (T2). To ensure the preciseness of the study and to minimize the influence of irrelevant variables, subjects who participated in both tests were finally adopted in this study, and the retrieved questionnaires were strictly screened to remove any with missing responses, straight-line responses (answering each question with the same answer, such as “111,111,111…” or “222,222,222…”), and pattern responses (following a certain artificial rule, such as “5,4,3,2,1,5,4,3,2,1…” or “5,5,5,4,4,4,3,3,3,2,2,2,1,1,1…”). The sample information is shown in Table 1.

**Table 1:** The sample information.

Basic information		Retained sample (*n* = 352)	Attrition sample (*n* = 124)
Gender, *n* (%)	male	207 (58.8%)	71 (57.3%)
	female	145 (41.2%)	53 (42.7%)
Age, *M* (*SD*)	age	18.83 (4.27)	18.52 (4.43)
Training age, *M* (*SD*)	training age	7.01 (3.25)	6.89 (3.46)
Sports grade, *n* (%)	master	134 (38.1%)	52 (41.9%)
	first-grade	148 (42.0%)	48 (38.7%)
	second-grade	70 (19.9%)	24 (19.4%)

Given the importance of sample size in the follow-up study, the study first analyzed the sample attrition rate, and the results showed that the sample attrition rate of the data was 26.1%; secondly, the difference test of the scores of the retained and attrition samples on basic information as well as T1athlete gratitude, T1athlete engagement and T1athlete burnout were administrated. The normality test showed that the retained and attrition samples showed a certain degree of skewed distribution on each basic information as well as T1athlete gratitude, T1athlete engagement and T1athlete burnout. Further tested by χ^2^ test and Mann–Whitney U test, the results showed that there was no significant difference between the retained and attrition samples on gender [χ^2^(1) = 2.86, *p* = 0.442), age (Z = −0.49, *p* = 0.580], training age (Z = −0.17, *p* = 0.643) and sports grade [χ^2^(2) = 2.07, *p* = 0.518], and there were also no significant differences in the scores of T1athlete gratitude (Z = −0.52, *p* = 0.403), T1athlete engagement (Z = −0.63, *p* = 0.703) and T1athlete burnout (Z = −0.50, *p* = 0.398), which together indicate that there was no structured attrition in the sample in this study.

### Measures

#### Gratitude questionnaire

The study used the Gratitude Questionnaire compiled by McCullough et al. (2002) and revised by Chinese scholar Chen (2013), a one-dimensional instrument consisting of five items, such as “As I have gotten older, I have become more appreciative of the support I receive from my coaches and teammates. They have become a part of my life” and “When I look back on my athletic career, I do not see much to be thankful for (reverse scoring question).” The five-point Likert scale was adopted, with higher scores indicating higher levels of athlete gratitude. The questionnaire Cronbach’s α coefficients for the two measurements in this study were 0.842 and 0.844, respectively, and the results of confirmatory factor analysis for the first measurement were: normed chi square(χ^2^/*df*) = 2.982, root mean square error of approximation (RMSEA) = 0.063, standardized root mean square residual (SRMR) = 0.045, Tucker-Lewis index (TLI) = 0.963, comparative fit index (CFI) = 0.954 and goodness of fit index (GFI) = 0.966. Following the recommendations of Schumacker and Lomax (2016), this scale was suitable for the sample studied.

#### Athlete engagement questionnaire

The study used the Athlete Engagement Questionnaire compiled by Lonsdale et al. (2007a) and others. The questionnaire includes four dimensions of confidence, vigor, dedication and enthusiasm, with 16 items, such as “I am full of passion in training and competition” and “I am fully committed to my sport.” The five-point Likert scale was adopted, with higher scores indicating higher levels of athlete engagement. The questionnaire also showed good reliability in the study of Chinese scholar Wang et al. (2014) and others. The total questionnaire Cronbach’s α coefficients for the two measurements of athlete engagement in this study were 0.869 and 0.875, respectively. The results of the confirmatory factor analysis for the first measurement were: χ^2^/*df* = 3.053, RMSEA = 0.066, SRMR = 0.043, TLI = 0.937, and CFI = 0.926, and GFI = 0.942. Following the recommendations of Schumacker and Lomax (2016), this scale was suitable for the sample studied.

#### Athlete burnout questionnaire

The study used the Athlete Burnout Questionnaire developed by Raedeke and Smith (2001) and others and revised by Chinese scholar Sun and Zhang (2013) and others, which includes three dimensions of emotional/physical exhaustion, reduced sense of accomplishment, and negative evaluation of exercise, with 15 items, such as “I feel physically and mentally exhausted by exercise,” “I do not care about sports performance as much as I used to.” The five-point Likert Scale was used, with higher scores indicating higher levels of athlete burnout. The total questionnaire Cronbach’s α coefficients for the two measurements of athlete burnout in this study were 0.858 and 0.862, respectively. The results of the confirmatory factor analysis for the first measurement were: χ^2^/*df* = 2.724，RMSEA = 0.067，SRMR = 0.042，TLI = 0.962，CFI = 0.966，GFI = 0.951. Following the recommendations of Schumacker and Lomax (2016), this scale was suitable for the sample studied. In addition, the three dimensions of this questionnaire have relative independence and their weights are different, so the formula of athlete burnout weighted total score established by Zhang and Zhang (2010) was adopted (*S*_weighted total score_ = *S*_emotional/physical exhaustion_ *0.21 + *S*_reduced sense of accomplishment_ *0.47 + *S*_negative evaluation of sports_ *0.32) to calculate the athlete burnout weighted total score.

### Data analysis

Data analysis was performed by software SPSS 20.0 and AMOS 23.0. The χ^2^ test and Mann–Whitney U test were used to test for differences between the retained and attrition samples; the Cronbach’s αcoefficient and the confirmatory factor analysis were used to test the reliability of the questionnaire; Harman’s single-factor test was used to test for common method bias; ANOVA was used to test for differences of the study variables in terms of gender, and sports grade; Biased correlation analysis was used to verify the correlation between the study variables; and structural equation modeling was used to verify the cross-lagged effects between the study variables.

## Results

### Common method variance test

The procedural control method and Harman’s single-factor test were used to examine possible common method bias in the administration of the test. On the one hand, selected a measurement instrument with high reliability and validity has been repeatedly confirmed by domestic and foreign scholars, and the anonymity and confidentiality of the survey were emphasized in the introductory part of the questionnaire; on the other hand, excluding basic and coded information, exploratory factor analysis of single-factor unrotation was conducted on all questions of both tests. Eight factors with characteristic roots greater than 1 were extracted from both administrations, and the variance rates of factor 1 were 25.342% (T1) and 26.018% (T2), which did not reach the critical value of 40% (Zhou and Long, 2004). According to the basis and principles of common method bias testing and control (Tang and Wen, 2020), it was shown that the common method effect was not significant for both administrations.

### The status quo analysis of athlete gratitude, athlete engagement and athlete burnout

As shown in Table 2, on five-point Likert scale, the total mean scores of T1 and T2 athlete gratitude reached 4.10 and 4.12, the total mean scores of T1 and T2 athlete engagement reached 3.98 and 4.07, and the total mean scores of T1 and T2 athlete burnout were 2.67 and 2.68, indicating that the overall level of athlete gratitude and athlete engagement of our athletes is high, and athlete burnout is at a medium level. The study also examined the differences among athlete gratitude, athlete engagement, and athlete burnout in gender and sports grade, and the results show that for both measurements, the gender main effects of athlete gratitude, athlete engagement and athlete burnout are not significant; the main effects of athlete gratitude (*F*_T1_ = 14.23, *p* = 0.000; *F*_T2_ = 15.11, *p* = 0.000) and athlete engagement (*F*_T1_ = 12.08, *p* = 0.000; *F*_T2_ = 12.35, *p* = 0.000) for the sports grade are significant for both, while the main effect of sports grade is not significant for athlete burnout; the interaction of gender and sports grade is not significant for athlete gratitude, athlete engagement and athlete burnout. In addition, the total mean scores of athlete gratitude level of master athletes (*M*_T1_ = 4.27, *M*_T2_ = 4.32) are significantly higher than those of 1 and 2-grade athletes (*M*_T1_ = 3.95, *M*_T2_ = 3.97) for both measurements in terms of sports grade; the total mean scores are also significantly higher for master athletes’ athlete engagement level (M_T1_ = 4.20, M_T2_ = 4.21) than for both 1 and 2-grade athletes (M_T1_ = 3.78, M_T2_ = 3.93).

**Table 2:** Main effects and interaction effects of each variable in gender and sports grade.

Variable	T1				T2			
	*M* (*SD*)	Main effect		Interaction effect	*M* (*SD*)	Main effect		Interaction effect
		Gender	Sports grade	Gender*sports grade		Gender	Sports grade	Gender*sports grade
Athlete gratitude	4.09 (0.47)	*F* = 0.17 *p* = 0.672	*F* = 14.23 *p* = 0.000	*F* = 0.53 *p* = 0.743	4.12 (0.43)	*F* = 0.16 *p* = 0.611	*F* = 15.11 *p* = 0.000	*F* = 0.50 *p* = 0.786
Athlete engagement	3.98 (0.67)	*F* = 0.21 *p* = 0.581	*F* = 12.08 *p* = 0.000	*F* = 0.60 *p* = 0.812	4.09 (0.55)	*F* = 0.19 *p* = 0.617	*F* = 12.35 *p* = 0.000	*F* = 0.58 *p* = 0.798
Athlete burnout	2.67 (0.31)	*F* = 0.33 *p* = 0.662	*F* = 0.47 *p* = 0.521	*F* = 0.71 *p* = 0.524	2.68 (0.30)	*F* = 0.32 *p* = 0.670	*F* = 0.45 *p* = 0.534	*F* = 0.70 *p* = 0.522

### Correlation analysis of athlete gratitude, athlete engagement, and athlete burnout

As in Table 3, bias correlation analysis was performed on athlete gratitude, athlete engagement and athlete burnout while controlling for variables such as gender and sports grade. Stable correlations show that there are significant correlations (*p* < 0.001) between T1 athlete gratitude and T2 athlete gratitude (*r* = 0.433), between T1 athlete engagement and T2 athlete engagement (*r* = 0.520), and between T1 athlete burnout and T2 athlete burnout (*r* = 0.479). Significant Pairwise correlations (*p* < 0.001) are showed between T1 athlete gratitude, T1 athlete engagement, and T1 athlete burnout; T2 athlete gratitude, T2 athlete engagement and T2 athlete burnout are also significantly pairwise correlated (*p* < 0.001). Taken together, this suggests that athlete gratitude, athlete engagement and athlete burnout satisfy intertemporal stability and simultaneous correlation over 1 year.

**Table 3:** Biased correlation analysis of each variable.

Variable	T1 athlete gratitude	T1 athlete engagement	T1 athlete burnout	T2 athlete gratitude	T2 athlete engagement	T2 athlete burnout
T1 athlete gratitude	1.000					
T1 athlete engagement	0.341^***^	1.000				
T1 athlete burnout	−0.385^***^	−0.501^***^	1.000			
T2 athlete gratitude	0.433^***^	0.398^***^	−0.279^**^	1.000		
T2 athlete engagement	0.346^***^	0.520^***^	−0.498^***^	0.321^***^	1.000	
T2 athlete burnout	−0.234^**^	−0.488^***^	0.479^***^	−0.406^***^	−0.399^***^	1.000

** denotes *p* < 0.01 and

*** denotes *p* < 0.001.

### Cross-lagged analysis of athlete gratitude, athlete engagement and athlete burnout

**Figure 1:**
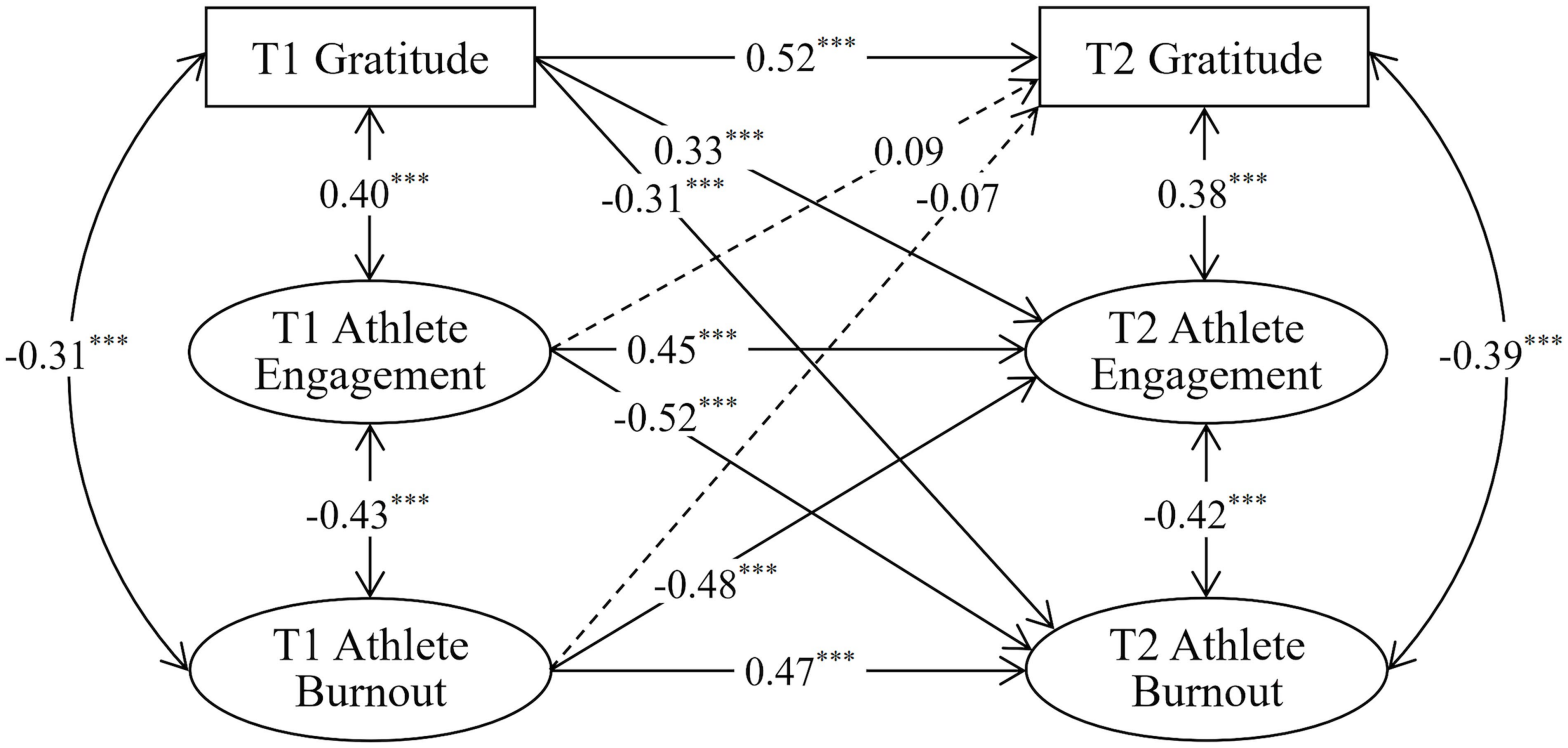
Cross-lagged model of athlete gratitude, athlete engagement and athlete burnout. ^***^p < 0.001. The values in the figure are standardized path coefficients.

As in Figure 1, the cross-lagged relationship between athlete gratitude, athlete engagement and athlete burnout were examined by adopting the structural equation model, setting the “T1 athlete gratitude→T2 athlete engagement” path coefficient as 1, and using the maximum likelihood method to test the fitness of the cross-lagged effect model. The model fit indexes show that normed chi square (χ^2^/*df*) = 3.577 (*p* < 0.001, *n* = 352); Tucker-Lewis index (TLI) = 0.965, comparative fit index (CFI) = 0.941, and goodness of fit index (GFI) = 0.956, all of which are greater than 0.9; RMSEA = 0.065, which is less than 0.08; and SRMR = 0.041, which is less than 0.05. Collectively, it shows that the cross-lagged effect model between athlete gratitude, athlete engagement and athlete burnout fit well and has acceptable fitness.

As shown in Figure 1, the asynchronous correlations of athlete gratitude, athlete engagement and athlete burnout were examined by model path coefficients, and the results show that (1) T1 athlete gratitude significantly predict T2 athlete engagement (*β* = 0.33, *p* < 0.001) and T2 athlete burnout (*β* = −0.31, *p* < 0.001); (2) T1athlete engagement significantly predict T2 athlete burnout (*β* = −0.52, *p* < 0.001), and T1 athlete burnout also significantly predict T2 athlete engagement (*β* = −0.48, *p* < 0.001); (3) T1athlete engagement and T1 athlete burnout are not significant predictors of T2 athlete gratitude (*β* = 0.09, *p* > 0.05; *β* = −0.07, *p* > 0.05). The combined model path coefficients and the inferred views of data from scholars Eisma et al. (2019) and Liu (2021) suggest that athlete gratitude is the causal variable for athlete engagement and athlete burnout, thus H3 and H4 are verified; athlete engagement and athlete burnout, on the other hand, are mutually causal, thus H3 and H4 are verified. In terms of the influence path, athlete gratitude can affect both athlete engagement and athlete burnout directly, indirectly affect athlete burnout through the mediating role of athlete engagement, and indirectly affect athlete engagement through the mediating role of athlete burnout.

### Supplementary analysis

A multi-group structural equation model was used to examine whether the relationship model between the variables shown in Figure 1 had gender equivalence and sports grade equivalence, with a free model (allowing free estimation of all parameters for male and female; allowing free estimation of all parameters for athletes at the master level and for athletes at I and II grades) and a restricted model (autoregressive and cross-lagged paths and factor loadings for male and female were limited to be equal; the autoregressive and cross-lagged paths and the factor loadings were limited to be equal for master, I and II-grades athletes). As shown in Table 4, the results indicate that the fit for both the restricted and free model on gender and sports grade had acceptable fitness, and with non-significant differences (ΔCFI _gender_ = 0.006, ΔCFI _sports grade_ = 0.005). Therefore, the cross-lagged model of the relationship between the variables shown in Figure 1 had gender equivalence and sports grade equivalence, and there are no significant gender differences or sports grade differences in the cross-lagged relationships between athlete gratitude, athlete engagement and athlete burnout. In addition, in order to exclude the influence of other variables on the relationship between athlete gratitude, athlete engagement and athlete burnout, the study introduced the variables of gender, sports grade, training age and age as control variables into the model based on the previous statistical analysis, and set gender, sports grade, training age and age to exogenous variables (T2 athlete gratitude, T2 athlete engagement and T2 athlete burnout) paths. As shown in Table 4, the results showed that the control variables are introduced to fit the model well and the results did not change substantially. Therefore, it can be concluded that the relationship between variables in this study is not due to the effect of other variables.

**Table 4:** Multi-group structural equation model fit index.

Model		χ^2^/*df*	RMSEA	SRMR	CFI	TLI	GFI	ΔCFI
Gender	restricted model	2.778	0.050	0.032	0.971	0.960	0.944	0.006
	free model	2.801	0.052	0.041	0.965	0.957	0.941	
Sports grade	restricted model	2.692	0.063	0.034	0.967	0.945	0.953	0.005
	free model	2.947	0.064	0.040	0.962	0.940	0.956	
Introducing the control variables into model		2.684	0.058	0.039	0.935	0.951	0.947	

## Discussion

### The status quo of athlete gratitude and athlete engagement

This study found that the overall level of athlete gratitude and athlete engagement in China was high, which is consistent with the findings of Ye et al. (2016a). Gratitude has been a traditional virtue of the Chinese people since ancient times, like the ancient motto of “Receiving drips of water when in need, and I shall return the kindness with a spring” and the old saying “it is not a gentleman who does not repay a kindness” have been widely spread and have a profound influence. In China’s unique “national system” of competitive sports management and training mode, athletes live, train and finish competition with their coaches and teammates all year round, and their growth is closely related to the guidance of coaches, support of parents and help of teammates, and cannot be separated from the financial security of the government and the concern of all walks of life. All this has laid a good foundation for the formation and cultivation of the character of athlete gratitude. In addition, Chinese sports administrative departments also pay high attention to the cultivation and education of athletes’ core values, such as promulgating *the Guidance on Further Strengthening Athletes’ Cultural Education and Athletes’ Protection*, *the Opinions on Cultivating and Practicing Socialist Core Values*, and holding a series of gratitude-themed educational activities, which provide important policy assurance and practical means for the cultivation of athlete gratitude (Dong et al., 2016).

This study also found that on the whole, Chinese athletes at master level had higher levels of gratitude and athlete engagement than athletes at I and II grades. In general, master athletes have more years of training, higher skill levels, and higher athletic performance than athletes at the I and II grades. Master Athletes spend more time and have closer relationships with their coaches and teammates, and receive more financial and social support, which help to motivate athletes to be more grateful, devote more time and energy to their sports, and thus show higher gratitude and athlete engagement tendencies in their daily lives and training.

### The predictive effect of athlete gratitude on athlete burnout and athlete engagement

Since gratitude has become the focus of positive psychology, the positive effects and value it generates have been widely recognized and confirmed by scholars (Dong et al., 2016). Based on broaden-and-build theory of positive emotions, cognitive-emotional stress model, intrinsic and extrinsic goal theory, and previous empirical findings, this study hypothesized that athlete gratitude significantly predicted athlete burnout and athlete engagement (H1, H2), and the analysis results of the tracking data supported the hypothesis, which is also consistent with Chen and Chang (2014), Wang et al. (2014), Ye et al. (2016a) and other studies’ analysis results of cross-sectional data.

On the one hand, gratitude can increase athletes’ psychological coping resources. It has been found that gratitude not only helps athletes develop optimistic attributional style, but also increases their subjective well-being, eliminates the adverse effects caused by negative emotions, and builds and maintains their psychological coping resources (Kong et al., 2021; Yang et al., 2021). Individuals with high gratitude tendencies view what they have as a gift, focus more on perceiving positive stimuli around them to obtain positive experience, and evaluate life more optimistically. Athletes with high gratitude tendencies can use these positive cognitive tendencies to ignite enthusiasm for training, energize for competition, and eliminate the undesirable effects of negative emotions, thus avoiding emotional/physical exhaustion (Wood et al., 2010; Yukhymenko-Lescroart et al., 2022). In addition, athletes with high gratitude tendencies are more appreciative of the support from companions, family, society, and the nation, and they are more resilient and motivated to respond to the expectations of all parties and to cope with stress and challenges with a positive attitude, which helps to avoid negative sports evaluation (Gabana et al., 2022).

On the other hand, gratitude can promote positive cognitive and behavioral changes in athletes. Gratitude is a powerful, positive life emotion that shapes our core beliefs, improves physical and mental health, boosts happiness, optimizes emotional and affective states, enhances coping capabilities, maintains spiritual peace and harmony, induces pro-social behavior, and has both individual and social value (Loi and Ng, 2021; Yang et al., 2021). The broaden-and-build theory of positive emotions also suggests that gratitude, as a positive emotion, can broaden an individual’s tendency to think and act, and that gratitude can also help individuals establish and strengthen social connections with others, thus forming good social networks (Emmons and McCullough, 2004). It has been found that gratitude can motivate adolescents to pursue and strive for goals and promote their school engagement, which in turn increases individual study engagement and academic achievement (Zainoodin et al., 2021; Zhen et al., 2021). Similarly, in sports field, athletes’ appreciation for significant others, society, and the nation would enhance athletes’ identification with training and competition, inspire their sense of responsibility, dedication, and motivate them to engage in training and competition with enthusiasm and energy (Wang et al., 2014).

### The mutual predictive effect of athlete engagement and athlete burnout

The results of cross-lagged analysis showed that T1 athlete engagement could significantly and negatively predict T2 athlete burnout, and T1 athlete burnout could also significantly and negatively predict T2 athlete engagement, which indicated that the higher the level of athlete engagement, the lower the level of athlete burnout; anyway, the lower the athlete burnout level is, the higher their athlete engagement level is, and the athlete engagement level and athlete burnout level can predict each other, validating H3 and H4 proposed by the study. Currently, most studies in sports field tend to consider athlete engagement as an important predictive variable of athlete burnout. Hodge et al. (2009) and others found that when athletes are able to use internal and external resources comprehensively to meet their independent, competence and relationship needs, and keep their cognitive, emotional and behavioral engagement in a high state, they can break through their self-handicapping, stimulate their athletic potential and gain more fluid experience. Zhang (2012) also argued that athlete engagement, as an important indicator of athletes’ positive psychological aspects, can reflect athletes’ positive and healthy psychological states, and is conducive to stimulating positive qualities such as optimism, resilience, sense of meaning and creativity in athletes, thus alleviating or inhibiting athlete burnout. Guo et al. (2021) also found that athlete engagement significantly predicted athlete burnout based on cross-sectional data from 753 Chinese adolescent athletes. In the field of education, the predictive role of student burnout on study engagement has begun to be explored. Xing et al. (2016) and Zhang et al. (2016) found that student burnout was predictive of study engagement, and students with lower student burnout would have higher levels of study engagement. In the field of organizational management, research on the relationship between work burnout and work engagement has transitioned from the initial phase of opposite validation to the current phase of integrated exploration. These two phases of research showed that work burnout and work engagement were opposite concepts in terms of core dimensions, but they were not “incompatible,” and the job demands-resources model and the dual-process model of positive and negative employee well-being was proposed to carry on an integration study on work burnout and work engagement (Wang and Qin, 2009). The research results in the fields of education and organizational management provide supporting evidence for the study of the relationship between athlete engagement and athlete burnout in the field of sports while also providing research directions for reference.

### Limitations and implications

There are limitations and challenges to the present study. Firstly, cross-lag design can provide information about the causal relationship between variables to a certain extent, but it lacked the evidence from scientific experiment. Future follow-ups should be continued to verify the stability of the relationship between athlete gratitude, athlete engagement and athlete burnout, even though experimental research to further verify the causal relationship among variables. Secondly, there are many factors affecting athlete burnout, this study only examined the effect of athlete gratitude, athlete engagement on athlete burnout. In the future, on the basis of extending the tracking time, more research variables (such as social support, parenting style) should be included to verify the stability of the relationship among athlete gratitude, athlete engagement, athlete burnout. In addition, future research should continue to tap the positive meanings of gratitude on athletes’ life, training and competition, such as the effects of gratitude on athletes’ social support, life satisfaction, mental toughness, “coach-athlete” relationship and so on. Further, theoretical and empirical research results in the field of organizational management should be drawn on to construct an integrated model of athlete engagement and athlete burnout research and conduct empirical exploration.

## Conclusion

This study drew the following conclusions. First, the overall level of athlete gratitude and athlete engagement was high in China. Chinese athletes at master level had higher levels of gratitude and athlete engagement than athletes at I and II grades. Second, athlete gratitude is a significant negative predictor of athlete burnout, and also a significant positive predictor of athlete engagement. Third, athlete engagement and athlete burnout are mutually causal and can be mutually predicted. Finally, athlete gratitude indirectly affects athlete burnout through athlete engagement, and also indirectly affects athlete engagement through athlete burnout.

## Data availability statement

The raw data supporting the conclusions of this article will be made available by the authors, without undue reservation.

## Ethics statement

Ethical review and approval were not required for the study on human participants in accordance with the local legislation and institutional requirements. Written informed consent to participate in this study was provided by the participants’ legal guardian/next of kin.

## Author contributions

ZG conceived and designed the research, undertook data analysis, and wrote the Chinese manuscript. ZG and MW wrote and supplement the English manuscript. JY and SC participated in the manuscript revision. ZG, YX and SL participated in the collection and sorting of data. All authors contributed to the article and approved the submitted version.

## Funding

This research was funded by the Fellowship of China Postdoctoral Science Foundation (Grant No.2021 M702543), Philosophy and Social Science Foundation of Shanghai (Grant No.2020ETY001), Education Science Research Project of Shanghai (Grant No.C2021024), and Scientific Research Fund of Young Teachers in Wuhan Sports University (Grant No.2022Z05).

## Conflict of interest

The authors declare that the research was conducted in the absence of any commercial or financial relationships that could be construed as a potential conflict of interest.

## Publisher’s note

All claims expressed in this article are solely those of the authors and do not necessarily represent those of their affiliated organizations, or those of the publisher, the editors and the reviewers. Any product that may be evaluated in this article, or claim that may be made by its manufacturer, is not guaranteed or endorsed by the publisher.
