# Supplementary material for: The associations among athlete gratitude, athlete engagement, athlete burnout: A cross-lagged study in China
Source: Front Psychol. 2022 Sep 29;13:996144. doi: 10.3389/fpsyg.2022.996144 (PMC9557925; doi:10.3389/fpsyg.2022.996144)
Supplement: Supplementary file 1 [file Table_1.DOCX]

# Supplementary Material

# (1) Gratitude Questionnaire

① I have so much in life to be thankful for

② If I had to list everything that I felt grateful for, it would be a very long list

③ When I look at the world, I don’t see much to be grateful for

④ I am grateful to a wide variety of people

⑤ As I get older, I find myself more able to appreciate the people, events, and situations that have been part of my life history

# (2) Athlete Engagement Questionnaire

① I believe I am capable of accomplishing my goals in sport

② I feel capable of success in my sport

③ I believe I have the skills/technique to be successful in my sport

④ I am confident in my abilities

⑤ I am dedicated to achieving my goals in sport

⑥ I am determined to achieve my goals in sport

⑦ I am devoted to my sport

⑧ I want to work hard to achieve my goals in sport

⑨ I feel energized when I participate in my sport

⑩ I feel energetic when I participate in my sport

⑪ I feel really alive when I participate in my sport

⑫ I feel mentally alert when I participate in my sport

⑬ I feel excited about my sport

⑭ I am enthusiastic about my sport

⑮ I enjoy my sport

⑯ I have fun in my sport

# (3) Athlete Burnout Questionnaire

① I’m accomplishing many worthwhile things in sport

② I feel so tired from my training that I have trouble finding energy to do other things

③ The effort I spend in sport would be better spent doing other things

④ I feel overly tired from my sport participation

⑤ I am not achieving much in sport

⑥ I don’t care as much about my sport performance as I used to

⑦ I am not performing up to my ability in sport

⑧ I feel “wiped out” from sport

⑨ I’m not into sport like I used to be

⑩ I feel physically worn out from sport

⑪ I feel less concerned about being successful in sport that I used to

⑫ I am exhausted by the mental and physical demands of sport

⑬ It seems that no matter what I do, I don’t perform as well as I should

⑭ I feel successful at sport

⑮ I have negative feelings toward sport

# (4) The measures to protect the privacy of the subjects

# This study adopted the following measures to protect the privacy of the subjects:

① Collect relevant information of subjects anonymously. Researchers and research team members cannot directly or indirectly identify subjects' names, ID numbers and IP information.

② After the data is recovered, it will be kept by the project leader (corresponding author of this study).

③ The computer that saves the data is protected by a password. The folder where the data is stored will also be password protected. Members participating in the project can access the data after declaring the reason for use and submitting an application in writing.

④ The relevant data of this study will be kept for 3 years after the completion of the project. Then, the project leader will supervise the data user to delete the data and not back it up. After all the research is completed, project leader will delete the data in a unified way.
